# Social Phobia in an Italian region: do Italian studies show lower frequencies than community surveys conducted in other European countries?

**DOI:** 10.1186/1471-244X-4-31

**Published:** 2004-10-15

**Authors:** Mauro Giovanni Carta, Maria Carolina Hardoy, Mariangela Cadeddu, Bernardo Carpiniello, Liliana Dell'Osso, Mario Antonio Reda, Hans-Ulrich Wittchen

**Affiliations:** 1 Division of Psychiatry, Department of Public Health, University of Cagliari, Italy; 2 Department of Psychiatry, Neurobiology, Pharmacology, Biotechnology, University of Pisa, Italy; 3 Division of Behavioural Sciences, Department of Neurological and Behavioural Sciences, University of Siena, Italy; 4 Institute of Clinical Psychology, Technical University of Dresden, Germany

## Abstract

**Background:**

The lifetime prevalence of Social Phobia (SP) in European countries other than Italy has been estimated to range from 3.5% to 16.0%. The aim of this study was to assess the frequency of SP in Sardinia (Italy) in order to verify the evidence of a lower frequency of SP in Italy observed in previous studies (from 1.0% to 3.1%).

**Methods:**

A randomised cross sample of 1040 subjects, living in Cagliari, in rural areas, and in a mining district in Sardinia were interviewed using a Simplified version of the Composite International Diagnostic Interview (CIDIS). Diagnoses were made according to the 10^th ^International Classification of Diseases (ICD-10).

**Results:**

Lifetime prevalence of SP was 2.2% (males: 1.5%, females: 2.8%) whereas 6-month prevalence resulted in 1.5% (males: 0.9%, females: 2.1%). Mean age at onset was 16.2 ± 9.3 years. A statistically significant association was found with Depressive Episode, Dysthymia and Generalized Anxiety Disorder.

**Conclusions:**

The study is consistent with findings reported in several previous studies of a lower prevalence of SP in Italy. Furthermore, the results confirm the fact that SP, due to its early onset, might constitute an ideal target for early treatment aimed at preventing both the accumulation of social disabilities and impairments caused by anxiety and avoidance behaviour, as well as the onset of more serious, associated complications in later stages of the illness.

## Background

Several epidemiological studies have attempted to describe the prevalence, socio-demographic characteristics, comorbidity, and severity of clinical manifestations of Social Phobia (SP). Quality of life and functional status of affected individuals have also been investigated [1]. Lifetime prevalence of SP in European societies other than Italy ranges from 3.5% to 16.0% [2]. As discussed in several excellent review papers, these rate differences were partly attributed to probable genetic or cultural factors [3]. Furthermore, major methodological differences (type of diagnostic criteria used, assessment tools, age of the sample) affecting the estimates have been demonstrated [1].

This study, part of an extended epidemiological investigation "Health in Sardinia," aimed to assess the prevalence rates of SP in Sardinia (Italy) in order to confirm the evidence of low SP prevalence rates ranging from 1.0% to 3.1% observed in previous research projects in Italy [4-6]. The study also intended to evaluate the treatments and to verify the comorbid psychiatric disorders in the identified people with Social Phobia.

## Methods

**Figure 1:**
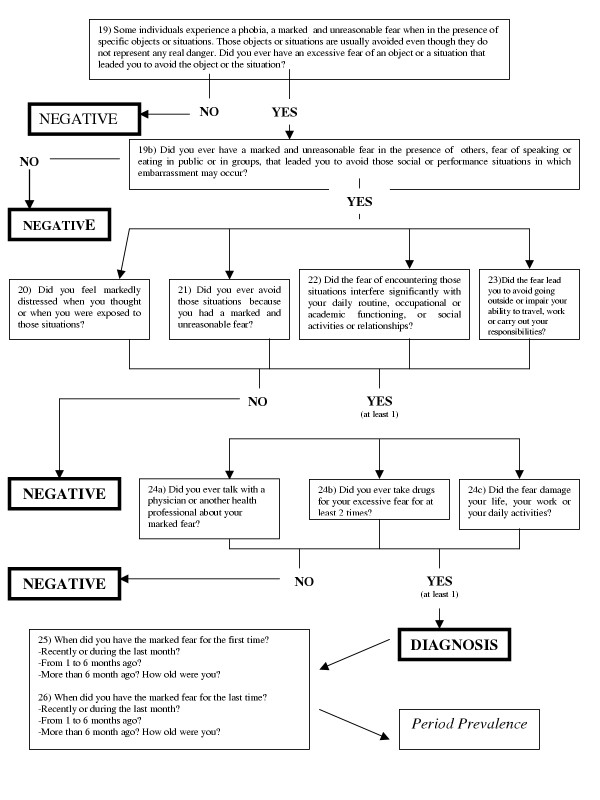
The Composite International Diagnostic Interview Simplified (CIDIS) [9, 10] algorithm for the diagnosis of Social Phobia.

The sample, which had already been examined and described in greater detail in a previously published study [7], consisted of 1040 subjects recruited throughout the island of Sardinia, Italy. 393 subjects came from the city of Cagliari, 344 from rural areas, and 303 from an industrial mining district, thus representing fairly well all socio-economic strata present on the island. The age distribution of the sample is shown in table 1, with age range from 18 to 89 years. 79.2% out of a total of 1313 subjects approached, agreed to take part in the study, 12.5% refused to participate, and 8.3% could not be traced; the final sample did not differ respect to the population of origin with reference to the variables applied in stratification (Table 1). All subjects were interviewed "face to face" by trained physicians using a Simplified version of Composite International Diagnostic Interview (CIDI) [8], hence the acronym CIDI "Simplified" (CIDIS) [9,10]. The version used in this study had been translated into Italian and back-translated into the original French language under blind conditions respect to the first translation, by a bilingual researcher; approval of the final version was obtained from the original authors [11]. The CIDI structured interview in its various versions currently represents the most widely used diagnostic tool in psychiatric epidemiological studies conducted on the general population [12]. The CIDIS is a highly structured tool made up of 5 sections which investigate respectively: Somatoform Disorders and General Medical Conditions, Anxiety Disorders, Depressive Disorders, Substance-Related Disorders (Alcohol-Related Disorders and Substance-Related Disorders) and Eating Disorders. The computer elaboration of data obtained through application of the CIDIS enables calculation of both "lifetime prevalence" and, for the preceding six months, a series of psychiatric disorders (those more frequently observed in the general population) according to the ICD-10 diagnostic system [13]. This interview moreover enhances identification of both the type of therapist referred to and treatment already used by each patient for his or her specific problem and definition of degree of impact of the problem on the subject's daily routine. An ad hoc computer algorithm ascertained the presence of the disorders according to ICD-10 criteria [13], both in the past six months and in the lifetime. The items of the CIDIS concerning Social Phobia and the related algorithm is reported in Figure 1.

**Table 1:** Percentage of subdivision according to age, sex, and marital status of the sample.

	N (1040)
MALES	461 (44.3%)
FEMALES	579 (55.7%)
AGE 18–24	146 (14.0%)
AGE 25–44	353 (33.9%)
AGE 45–64	310 (29.8%)
AGE >64	231 (22.2%)
UNMARRIED	370 (35.6%)
MARRIED	571 (54.9%)
WIDOWED/SEPARATED/DIVORCED	99 (9.5%)

## Results

Estimates of lifetime SP prevalence of 2.2% were found (males: 1.5%, females: 2.8%), with no statistically significant difference between the sexes (χ^2 ^= 1.2, 1DF, P = 0.25). 6-month prevalence rates were lower (total: 1.5%, males: 0.9%; females: 1%; no significant difference between the sexes, χ^2 ^= 1.6, 1DF, P = 0.13). Table 2 compares results obtained in this study with those of the major research projects carried out in Europe and the USA [4,5,14-23]. Table 3 illustrates the lifetime prevalence according to age and sex, and Table 4 shows the 6-month prevalence according to age and sex. An increased frequency of SP among the younger age groups was observed, although distribution in males resulted as being less homogeneous. In no case statistical significance was reached. Lifetime prevalence rates of SP respect to marital status of subjects studied were as follows: 3.0% among the unmarried; 1.2% among married subjects; and 3.5% among the divorced, separated and widowed (χ^2 ^= 5.8, 1DF, P = 0.06). Mean age at onset was 16.2 ± 9.3 years.

**Table 2:** Lifetime prevalence of Social Phobia in the general population of Europe and USA.

Country	Reference	Diagnostic criteria	N	Male	Female	Total
Italy	Faravelli et al., 1989 [4]	DSM-III-R	1110			1.0
Switzerland	Wacker et al., 1992 [16]	DSM-III-R	470			16.0
		ICD-10				9.6
ECA (USA)	Schneier et al., 1992 [15]	DIS	18571	2.0	3.1	2.4
Iceland	Lindal and Stefansson, 1993 [32]	DSM-III	862	2.5	4.5	3.5
Switzerland	Degonda and Angst, 1993 [21]	DSM-III	591	3.1	5.7	4.4
NCS (USA)	Kessler et al., 1994 [17]	CIDI	8098	11.1	15.5	13.3
France	Lepine and Lellouch, 1995 [19]			2.1	5.4	4.1
Germany	Wittchen et al., 1998 [23]	DSM-IV	3021	2.2	4.8	3.5
Italy	Carta and Rudas, 1998 [6]	CIDI	783	1.7	4.6	3.1
Spain	Arillo et al., 1998 [cited in 33]	DIS	237		8.9	
Netherlands	Bijl et al., 1998 [20]	DSM-III-R	7076	5.9	9.7	7.8
France	Lépine and Pélissolo, 1999 [22]	DSM-IV				7.3
Italy	Faravelli et al., 2000 [5]	FPI/CIDI	2355	1.9	4.0	3.1
Italy	Carta et al., 2002 [7]	CIDI	1040	1.5	2.8	2.2

**Table 3:** Lifetime prevalence N (%) of Social Phobia according to age and sex.

Age	Male	OR	Female	OR	Total	OR
<25	1 (1.3)	0.9	4 (5.0)	2.3	5 (3.4)	1.7
25–44	4 (2.5)	2.5	4 (2.1)	0.7	8 (2.2)	1.1
45–64	1 (0.7)	0.4	4 (2.4)	0.8	5 (1.6)	0.6
>65	1 (1.1)	0.7	4 (2.8)	1.1	5 (2.2)	0.9

Male according to age χ^2 ^with Yathes correction = 1.6, 3 df, p = 0.89; female according to age χ^2 ^with Yathes correction = 2.2, 3 df, p < 0.71; total sample according to age χ^2 ^with Yathes correction = 1.8, 3 df, p = 0.82

**Table 4:** Six month prevalence N (%) of Social Phobia according to age and sex.

Age	Male	OR	Female	OR	Total	OR
<25	1 (1.5)	1.8	3 (3.7)	2.0	4 (2.7)	2.1
25–44	1 (0.6)	0.6	3 (0.7)	0.5	4 (1.1)	0.6
45–64	1 (0.7)	0.7	2 (1.1)	0.5	3 (0.9)	0.5
>65	1 (1.1)	1.4	4 (2.8)	1.4	5 (2.1)	1.6

Male according to age χ^2 ^= 0.6, 3 df, p = 0.98; female according to age χ^2 ^with Yathes correction = 3.4, 3 df, p = 0.44; total sample according to age χ^2 ^with Yathes correction = 3.4, 3 df, p = 0.44

During the week prior to the study, 8 (50.0%) out of the 16 subjects who had been diagnosed with SP over the past six months had been taking low doses of anxiolytics (less than the equivalent of 2 mg of Lorazepam). 3 (18.7%) were on antidepressants, one of whom (6.2%) at non-therapeutic doses, and 1 subject (6.2%) was undergoing cognitive behavioural psychotherapy. The remaining 6 subjects (37.5%) were not on any treatment.

Six out of the 10 treated subjects (60%) were being supervised by their general practitioners (GP), 1 (10%) by a neurologist and 2 (20%) by psychiatrists. All subjects presented some degree of comorbidity with Depressive Episodes (DE), Panic Attack Disorder (PAD), and agoraphobia (AP). Only 1 subject (10%) was undergoing cognitive behavioural therapy with a psychologist/psychotherapist.

Table 5 illustrates the rate of comorbidity with major psychiatric disorders observed in the general population, as well as the degree (OR) of associated disorders observed with regard to frequency reported for the latter in populations not affected by SP. A statistically significant difference was revealed for association with DE, Dysthymia (DD), and Generalized Anxiety Disorder (GAD). In spite of their increased frequency among patients affected by SP, disorders such as PAD and Specific Phobia do not represent a statistically significant association. The mean age at onset of comorbid DE was 6.5 ± 6.6 years subsequent to onset of SP, whereas GAD was manifested at a mean of 4.3 ± 7.8 years later.

**Table 5:** Lifetime comorbidity of Social Phobia.

	N (%)	OR	χ^2^
Depressive Episode (DE)	9 (39.1)	4.3	11.1*
Dysthymia (DD)	5 (21.7)	7.1	14.1*
Generalized Anxiety Disorder (GAD)	10 (43.4)	6.5	20.9*
Panic Attack Disorder (PAD)	2 (8.7)	3.3	1.1
Specific Phobia	1 (4.3)	8.6	1.6

*p < 0.001

## Discussion

Several epidemiological studies carried out in Europe (Switzerland [16,21], France [19,22], Germany [23]) and in the USA [15,17], recently reviewed by Furmark [2], suggest that SP is one of the more frequently observed anxiety disorders in the general population in Western countries. However, frequency rates reported in the various studies differ from country to country and according to time and evaluation methods used. Indeed, the two American studies [15,17] carried out at an interval of approximately 15 years, illustrate distinctly contrasting results, and it is hard to establish what factors really determine variance in findings.

The present study is consistent with the tendency towards rather low lifetime prevalence rates of SP observed in other Italian research projects. If we take into account only those European researches that adopted ICD-10 diagnosis, our results seem to indicate a lower frequency than a study carried out in Formentera, Spain (lifetime prevalence of 2.8% against 8.9% in females [cited in [33]]) and Basel, Switzerland (lifetime prevalence in the total sample 2.2 against 9.6% [16]). However, lower frequencies emerged also in Italian surveys conducted using different methods [5,7]. The Italian studies were carried out over a considerable period of time and in two distinct areas: the Florence area [4,5] and Sardinia [[6], the present study]. It is therefore quite likely that the lower frequencies observed may be the result of an effectively reduced vulnerability of Italians to SP. The results of this study can indeed be assumed as being influenced by several cultural variables – the genetic diversity of the two populations examined, as well as the considerable genetic variance of the Sardinian population respect to other European populations [24,25].

Findings for six-month prevalence show lower frequencies than those evidenced in the recent studies: 1.5% against 4.0% in Iceland [26] and against 4.0% in Munich [14]. Our rates are lower than E.C.A. findings: 2.7% in Duke [27] and 2.2% in Baltimore, New Haven, and Saint Louis [28]. The 6-month prevalence rates obtained in Sardinia are similar only to data reported for Edmonton (1.2%) from a survey carried out more than 15 years ago [29] and published in 1994 [30]. However, the prevalence data emerging from this study further justify the considerable interest shown in this disorder from both a medical and a social point of view. This research confirms the fact that onset of the illness occurs primarily during childhood and adolescence [31], thus underlining the suitability of the condition as a candidate for early treatment aimed at preventing both the slow accumulation of social disabilities and impairment caused by anxiety and by avoidance behaviour, as well as the onset of more serious complications (e.g., DE or GAD), which may be manifested many years after onset of SP. Indeed, subjects affected by social phobia presented a high risk of comorbidity with both the latter disorders and DD [1] .

It should be underlined that 60% of subjects undergoing treatment (not all affected subjects) chose their general practitioner (GP). This view is corroborated by the fact that those patients treated by a psychiatrist invariably presented comorbidity with DE, PAD, and AP, thereby representing the more severely affected from a psychopathological point of view. Overall however, the low rate of patients with SP treated with first line-treatments is alarmingly low.

In the future, serious attempts should be made to improve the GPs' abilities to recognise SP in order to prevent the use of inappropriate treatments, such as insufficient doses of benzodiazepines, which may be linked to the physician's incorrect diagnosing of the disorder. Due to the fact that subjects affected by SP most frequently refer to their GP, the importance of preventing SP as opposed to other types of disorders, as well as the markedly incapacitating nature of SP reinforce this necessity for a better training of GPs.

It is however mandatory to briefly acknowledge some potential limitations of this study. First, the number of cases and the sample size is too small to allow firm conclusions to be drawn concerning the true rate and the degree to which they might actually differ from previous studies with higher estimates. Secondly, differences in the assessment strategy might have resulted in a diminished comparability.

## Conclusions

The study is consistent with findings reported in several previous studies of a lower prevalence of Social Phobia in Italy and confirms the fact that onset of the illness occurs primarily during childhood and adolescence. Furthermore, the results confirm the fact that SP, due to its early onset, might constitute an ideal target for early treatment aimed at preventing both the accumulation of social disabilities and impairments caused by anxiety and avoidance behaviour, as well as the onset of more serious, associated complications in later stages of the illness, or many years after onset of SP.

## Competing interests

The authors declare that they have no competing interests.

## Authors' contributions

MGC participated in the design of the study, performed the statistical analysis and drafted the manuscript. UHW participated in the statistical analysis and drafted the manuscript. MCH, MC, BC, LDO, MAR conceived of the study, and participated in its design and coordination. All authors read and approved the final manuscript.

## Pre-publication history

The pre-publication history for this paper can be accessed here:


